# Boiled yam end‐user preferences and implications for trait evaluation

**DOI:** 10.1111/ijfs.14707

**Published:** 2020-07-25

**Authors:** Laurenda Honfozo, Laurent Adinsi, Alexandre Bouniol, Sounkoura Adetonah, Lora Forsythe, Ulrich Kleih, Joseph D. Hounhouigan, Geneviève Fliedel, Noël H. Akissoe

**Affiliations:** ^1^ Faculté des Sciences Agronomiques Université d'Abomey‐Calavi 01 BP 526 Cotonou Bénin; ^2^ CIRAD UMR QUALISUD 01BP52 Cotonou Bénin; ^3^ Qualisud Univ Montpellier CIRAD Montpellier SupAgro Univ d'Avignon Univ de La Réunion 73 avenue JF Breton, Montpellier Cedex 5 Montpellier 34398 France; ^4^ International Institute of Tropical Agriculture (IITA)‐Benin 08 BP 0932 Cotonou Bénin; ^5^ Natural Resources Institute (NRI) University of Greenwich Central Avenue, Chatham Maritime Kent ME4 4TB UK; ^6^ CIRAD UMR QUALISUD F‐34398 Montpellier France

**Keywords:** Check all that apply, consumer testing, gender, just about right, quality characteristics, sensory descriptors, surveys

## Abstract

This study aimed to establish the quality characteristics of raw and boiled yam by involving stakeholders along the food chain using a methodology that includes a state of knowledge review, focus group discussion and individual interviews, participatory processing diagnosis with processors and consumer testing. Predictive characteristics of yam for producing a high‐ and low‐quality boiled yam were related to morphological or physicochemical characteristics: peeled yam discoloration and mucilage content being negatively appreciated while the ease of peeling, viscous state of cooking water and the ease of breaking yam into pieces positively valued. High‐quality boiled yam should be white or yellowish, sticky to the fingers, nonfibrous, easy to chew, crumbly/friable, with a sweet taste and a good smell. The overall liking of boiled yam is greatly penalised by a too dark colour, hard to the touch, no sweet taste and no friability while eating.

## Graphical abstract

Final quality characteristics of boiled yam as a resultant of stakeholders' demand and processing ability of raw yam.

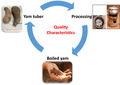

## Introduction

Yam (*Dioscorea spp*) is a major staple food in the tropics and subtropics of Africa, Southeast Asia and the Caribbean regions. It is a source of dietary calories and nutrients and contributes to household income (Egesi *et al*., 2003; Liu *et al*., 2007). The most important species of yam in West Africa are white yam (*Dioscorea rotundata*) and the African complex *D. cayenensis–D. rotundata*, also referred to as Guinea yam (Loko *et al*., 2015). The bulk of edible yam is produced in the *yam belt* of West and Central Africa, which accounts for approximately 97.2% world production with 73.02 million tons produced in 2017 (FAOSTAT, 2019). Benin is the fourth largest producer of yam in Africa behind Nigeria, Côte d'Ivoire and Ghana. Benin leads consumption in West Africa at 395 kcal per capita per day, followed by Côte d'Ivoire (331 kcal), Ghana (314 kcal), Togo (234 kcal) and Nigeria (204 kcal) (Laly *et al*., 2019).

Yam is consumed in various forms, including products prepared from raw tubers (pounded, boiled, fried or stewed) and processed in dried flour (yam chips). Boiled yam is considered an important food product that can be consumed at all meals and as a snack. It is consumed in the rural and urban areas of Benin as a street food. Boiled yam pieces are prepared by peeling, washing, slicing and cooking, which is performed by immersing the yam pieces in boiling water or by steaming. In the ‘yam belt’, users select yam varieties depending on their end‐use suitability: for pounding, boiling, drying (Zannou *et al*., 2007; Loko *et al*., 2015).

Several varieties of yam are grown and used to make different products. Laboko is recognised as the most preferred yam variety for pounded yam (Akissoe *et al*., 2011), and Kokoro for dried yam flour (Akissoe *et al*., 2003). For boiled yam, all varieties can, by tentative effort, be used, but all of them are not really acceptable for boiling and consuming (Loko *et al*., 2015). Some characteristics required for raw yam (tuber size, appearance, freshness, ease to use) (Barlagne *et al*., 2017) and boiled yam (white colour, crumbly and sweet taste) have been reported in literature (Naziha, 2002; Egesi *et al*., 2003; Otoo & Asiedu, 2009; Ranaivosoa *et al*., 2010), that have conducted individual interviews or focus group discussions, involving either farmers, processors or consumers.

Quality characteristics' demand of crop and product are specific to each type of stakeholders in a food chain. Previous research has developed methodologies that capture information from all food chain stakeholders on many food crops. Dedehouanou *et al*. (2017) argued that participatory evaluation of maize varieties along the food chain was required to ensure objective information and suggested that spatial and gender representativeness have to be considered in the choice of different users during consultation. A new approach was developed by Fliedel *et al*. (2016) for better assessing the adoption of new cassava genotypes, in a view of providing information to breeders early in varietal improvement programmes. It involved several successive steps such as qualitative surveys all along the food chain to identify quality criteria of a good cassava crop and product, effective participation of processors to identify the ability of genotypes to make a good product, and a ‘all‐in‐one’ method coupling hedonic test, JAR ‘Just All Right’ test, CATA ‘Check All That Apply’ questions to assess the acceptability and preferences of products by a large number of consumers. Furthermore, Forsythe *et al*. (2021) reported an interdisciplinary and participatory methodology to assess user acceptability of root, tuber and banana varieties, with the ultimate aim of informing breeding programmes in trait selection. This study aimed to identify the quality characteristics (morphological, postharvest, physicochemical, technological and sensory characteristics) of raw and boiled yam, required by producers, traders, processors and consumers in Benin, and how they influence varietal preferences.

## Materials and methods

### General approach

For the study, the multidisciplinary methodology as described by Forsythe *et al*. (2021) was used. This includes a state of knowledge review, gendered food mapping, participatory processing diagnosis and consumer testing, that will produce a list of prioritised quality characteristics for boiled yam. It is expected to translate these quality characteristics into physical or chemical components, for breeders to be used in their breeding programme in view of developing new varieties adopted by food chain stakeholders.

### Gendered food mapping in rural communities

The survey was carried out in eight rural communities of central Benin belonging to the Districts of Dassa‐Zoumé and Djidja. A survey was undertaken using the multidisciplinary methodology described by Forsythe *et al*. (2021). Participants were yam farmers, processors/cookers and consumers of boiled yam, who were selected randomly from different localities. In total, sixteen focus group discussions (FGDs) were held (eight men and eight women). In addition, eighty individual interviews including sixty‐four community members, eight market leaders and eight community leaders were completed.

Interviews were conducted in French and local languages. The questionnaire focused on yam varieties, processing steps to make boiled yam and quality characteristics of raw and boiled yam. It identified the quality characteristics valued by different user groups, prioritised them by importance and examined differences in by gender and region.

### Processing diagnosis of raw yam and quality characteristics

This study was carried out in Bohicon, Zou District, a small urban centre in southern Benin. Six skilled processors with at least 10 years of processing activity were recruited to make boiled yam using their own cooking methods (boiling or steaming). Six varieties selected for their contrasting characteristics, with five *Dioscorea rotundata* varieties (Laboko, Kodjèwé, Gnidou, Kpaina, and Deba) and one *Dioscorea alata* variety (Kpètè) (Table 1). Tubers were harvested 9 months after planting. The six varieties were coded with a random three‐digit number and presented to each processor in random order. Each processor received one to three tubers of each variety, weighing between 1.0 and 3.5 kg per variety. Key parameters such as duration, temperature and mass balance were determined at each step. Each processor was asked to describe the quality characteristics (the appearance of raw yam, peeling and cooking ability) of the yam at each step of the process (appearance, peeling and cooking ability of raw yam), and assess the final product (sensory and physical characteristics of boiled yam pieces).

**Table 1:** Description of yam varieties studied

Variety	Species	Nb. Harvest/year	Tuber characteristics
Laboko	*D. rotundata*	2	Big size, long, cylindrical, smooth
Kpaina	*D. rotundata*	1	Small size, smooth
Deba	*D. rotundata*	1	Small size, smooth
Kodjèwé	*D. rotundata*	2	Big size, long, often high thorny
Gnidou	*D. rotundata*	2	Regular, long, cylindrical, smooth
Kpètè	*D. alata*	1	Big size, smooth

Source: Dansi *et al*. (1997).

### Key processing parameters

The dry matter content (DMC) was determined by oven drying at 105 °C to constant weight according to AOAC method (1984). Quantitative processing parameters (duration, temperature, mass balance) were measured at each step of the process. The mass balance was determined at each step according to the method reported by Badoussi *et al*. (2015).

### Consumer testing in rural and urban areas

Consumer testing was carried out with 301 consumers interviewed randomly in different locations: 129 in eight rural communities, fifty‐two in Bohicon, a small urban town, and 120 in the city of Cotonou and its neighbourhood. Consumers were 18–72 years old and included 57.8% males and 42.2% females. Five out of the varieties that were used in the previous processing diagnosis were processed into boiled yam pieces by one skilled processor at each location following the same procedure. In order to maintain a warm temperature for the boiled yam samples for the consumer testing activity, batches of boiled yam pieces were made and stored for 5 min in an insulated container (60–65 °C). Each boiled yam samples (50–65 g) made from each yam variety were presented consecutively to each consumer in a plastic glass coded with a 3‐digit label. Mineral water was available for cleaning palate between samples testing. Consumers were asked to evaluate overall liking of each boiled yam using a 9‐point hedonic scale (1 = ‘Extremely dislike’, 9 = ‘Extremely like’). The intensity of five quality characteristics identified as important during previous surveys (colour, stickiness in fingers, hardness in hand, friability in mouth and sweet taste) was evaluated using the 3‐point ‘just about right’ (JAR) test (1 = ‘TW: too weak’ 2 = ‘JAR: just about right’ and 3 = ‘TS: too strong’). Each boiled yam samples were described with a Check All That Apply (CATA) table including fifteen sensory characteristics (slightly yellow, white, dirty white, pinkish, sticky, easy to chew, no fibres, friable/tender, hard in the mouth, easy to break with the hand, slightly bitter, tasteless, sweet taste) and five emotional terms (attractive, good smell of yam, good to eat, bad taste, unpleasant to eat). All the terms were selected from aforementioned surveys and then randomised between and within respondents (Ares & Jaeger, 2013).

### Data analysis

Qualitative data were analysed based on verbatim transcription in Excel, followed by descriptive analysis and prioritisation of characteristics weighing (Forsythe *et al*., 2021). The robustness of characteristics cited by the respondents was checked by revisiting the similarity/synonymy between verbatim words used by respondents or transcribed by different interviewers (variation in vocabulary). Important and robust characteristics were selected and prioritised according to the ranking order or to the citations weighting, where it is necessary. In the case of the citations weighting, if the sum is more than ten, the quality criterion is considered in the definition of the product profile. Where it is appropriate, a chi‐square test or Fisher's exact test was performed on number of citations in links with gender.

For participatory processing diagnosis, key quantitative processing parameters (duration, temperature, mass balance) were submitted to descriptive statistics.

The overall liking scores were submitted to variance analysis (anova) and Tukey's test (*P*‐value < 0.05). For CATA data, Cochran's *Q* test was carried out on the frequency of citations of each characteristic followed by correspondence factorial analysis (CFA). JAR test data were analysed for each characteristic by counting the percentage of respondents who evaluated each boiled yam sample as JAR ‘just about right’ or TW ‘too weak’ or TS ‘too strong’. Penalty analysis as defined by Gere *et al*. (2017) was performed to identify potential directions for consumers demand on the basis of the five selected sensory characteristics. JAR and overall liking scores of all boiled yam samples were combined to determine important mean drops in overall liking when the characteristics were cited TW or TS by at least 20% of consumers (Pareto principle) (Meullenet, Xiong *et al*., 2007). All statistical analyses were performed using XLSTAT version 2015.6.08 software (Addinsoft, Paris, France).

## Results and discussion

### Food crops prioritised by gender and region: importance of yam compared to other food crops

Table 2 shows that the most important food crops grown in the communities were maize, cassava, yam, soya and groundnut. The top three crops were maize, yam and cassava, maize being the most important crop irrespective of gender and region. Starchy crops (maize, yam and cassava) were positioned as priority crops while legumes (source of proteins) such as soya and groundnuts are used mainly as cash‐crops, suggesting that the rationale for growing a crop in both communities and for both men and women is above all for household consumption and sale (Tables 2 and 3). Regarding the top three crops, no spatial (regional) difference was observed in ranking. However, across regions, women ranked yam as the second most important crop and cassava as third, and vice versa for men. The first position of maize in both regions and across gender can be explained considering that maize is both a cash crop and important for household consumption. In addition, it can be stored for longer time than any other crop, so it is available for common use at any time (Nago *et al*., 1997). According to a women's FGD, yam is preferred to other crops because it can be harvested after 6 months from planting and easily stored for home consumption. Men's FGD argued that they prefer cassava because they can sell it all along the year to raise cash, and as reported by Saranraj *et al*. (2019), cassava provides security against famine mainly because of its staggered harvest.

**Table 2:** Main food crops and yam varieties grown in rural communities ranked by gender and region based on perception of importance (*n* = 16 focus group discussions)

Importance	Women^a^	Men^a^	Dassa^b^	Djidja^b^
	Main food crops grown			
1st	Maize	Maize	Maize	Maize
2nd	Yam	Cassava	Yam	Yam
3rd	Cassava	Yam	Cassava	Cassava
4th	Soya	Groundnuts	Groundnuts	Soya
5th	Groundnut	Soya	Soya	Groundnut

	Main Yam varieties grown			
1st	Laboko	Laboko	Laboko	Kokoro
2nd	Kokoro	Moroko	Moroko	Laboko
3rd	Moroko & Kpètè	Kokoro	Tchewere	Gnidou
4th	Gnidou	Gnidou	Kokoro	Kpètè
5th	Aklachi	Aklachi	Yanrambo	Aklachi

^a^ Across men and women.

^b^ Across districts.

**Table 3:** Reasons for growing yam varieties (% of respondents, *N* = 80)

Varieties	Reasons why preferred	Dassa (*n* = 40)	Djidja (*n* = 40)	Men (*n* = 29)	Women (*n* = 51)
Laboko	Good income	45	25	45	29
	Good to pound	27	22	24	25
	Earlier harvest (6 months)	32	10	34	14
	High yielding	15	2	14	6
	Domestic consumption	10	12	14	10
Kokoro	Good to pound	10	22	17	16
	Good income	5	15	10	10
	Processing into Amala	5	12	14	6
	Good to boil	2	12	7	8
	Domestic consumption	0	15	14	4
Gnidou	Big size	0	20	7	12
	Good income	2	20	17	8
	Good to boil	7	10	10	8
	Domestic consumption	0	7	7	2
	Good to fry	5	2	0	6
Moroko	Good income	20	0	10	10
	Good to pound	17	0	10	8
	Harvest before 10 months	15	0	7	8
	Domestic consumption	10	0	7	4
	High yielding	7	0	7	2
Tchewere	Good to pound	10	0	7	4
	Harvest before 10 months	7	0	7	2
	Domestic consumption	5	0	3	2
	Good income	5	0	3	2

Laboko, Kokoro and Moroko yam varieties are the most important varieties irrespective of gender and region under study (Table 2), in agreement with previous work (Dansi *et al*., 1997; Zannou *et al*., 2007). Laboko was ranked the most important variety by both men and women as it has the best quality for preparing many yam dishes based on processing ability and sensory traits. Laboko was seen as conferring social prestige since farmers stated: *Planting Laboko is a mark of respect and this confers to farmers the status of resource person for decision‐making that affects the development of the community*. Although gender difference was not significant (*P*‐value of 0.15 for chi‐square test) as far as all varieties are concerned, we observed that high proportion of women prefer Kokoro for its shelf life and quality of dried yam chips (size, ease to dry, colour and texture of derived dough), whereas men prefer Moroko for its high market value. The ranking of varieties differed by region, in relation to the quality requirements (essentially pounding ability and sensory) of local consumers: Moroko, Tchewere, Yanrambo for Dassa region, and Gnidou, Kpètè, Aklachi for Djidja. In the Dassa region, pounded yam is preferentially prepared year‐round. In addition to Laboko, and similar to Dassa region, Yanrambo also provided excellent pounded yam as reported by Zannou *et al*. (2007).

The motives/rationale of planting the top five preferred yam varieties can be grouped according to several criteria depending on varieties, region and gender (Table 3): agronomic performance (high yielding, big size, and early varieties), cash‐providing, and/or processing ability (good to pound, good to boil). All varieties are used both for sale and for home consumption, in agreement with Zannou *et al*. (2007) who pointed out that about half of harvested yam is designated for home consumption and the other half for sale. Thus, the choice of variety is influenced by variety type and regional/spatial differences in preference.

### Boiled yam processing diagnosis: key processing parameters

**Figure 1:**
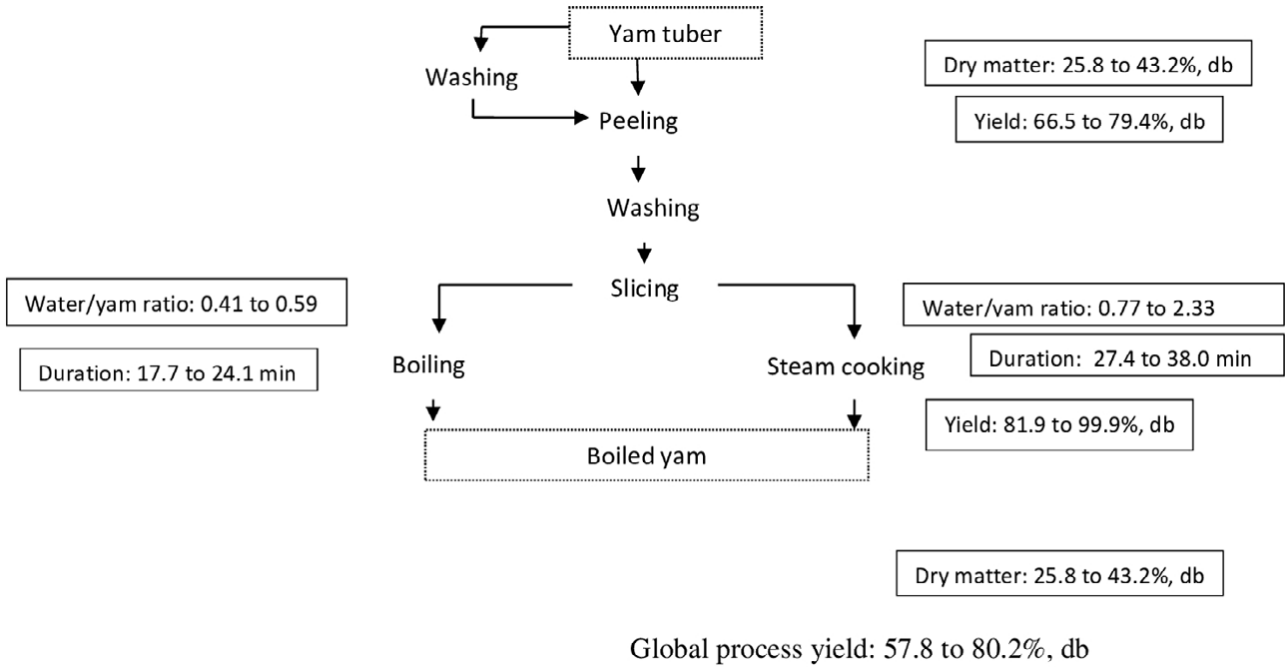
Flow diagram for making boiled yam and key processing parameters.

Boiled yam is obtained through a very simple process, including peeling, washing, slicing and boiling or steaming the yam pieces until the tuber cores become soft (Fig. 1). Processors prefer boiling yam pieces when the quantities are small for home consumption, and steaming when they have large quantities to sell, mainly because steam cooking better preserves the integrity of yam tubers all day long during selling.

The water‐to‐yam ratio during boiling was lower than that during steaming, ranging between 0.41 to 0.59 vs. 0.77 to 2.33, respectively. Steaming time was longer than that of boiling, with values in the range of 27.4–38.0 min vs. 17.7–24.1 min, respectively. Dry mater content of raw yam pieces was similar to that of boiled yam pieces, ranging from 25.8 to 43.2 g per 100 g, db. These results are in accordance with previous work that reported a positive relation between dry matter content of raw and cooked yam pieces (Akissoe *et al*., 2011). These authors pointed out that dry matter content of cooked yam decreased slightly for about 4 g per 100 g (wb) during cooking and can be predicted by the uncooked value.

### Raw yam quality characteristics

Quality characteristics of raw yam were collected during surveys and processing diagnosis and then disaggregated by gender (Table 4). They were morphological characteristics (appearance of skin, size and form of tuber, appearance of inner peel) and technological characteristics (skin colour during peeling, viscous exudate/mucilage). In general, women cited more characteristics than men, with more variation within those characteristics. Characteristics associated with high‐quality raw yam were cited about twice compared to those indicating poor‐quality raw yam. Ten out of twelve characteristics referring to high quality of raw yam are common for women and men, even if they were cited at a different frequency. Surveys and processing diagnosis data revealed that most of the characteristics were exclusively and separately grouped as high‐ or low‐quality types (Table 4), except for rough/shaggy/humped skin, which crosses groups. Test‐peeling with fingernails before buying tubers in the market provides reliable information on the raw yam quality, such as colour and water content. Significant differences were evidenced between men and women citations (chi‐square *P*‐value < 0.05) for some characteristics such as rough/shaggy/humped peel, brown skin, big tuber and presence of insects (Table 4). Some of the characteristics (big tuber, smooth, presence of rootlets, skin discolouration) matched up with previous work (Naziha, 2002; Otoo & Asiedu, 2009; Barlagne *et al*., 2017) while others related to the form of tuber (pointed tip, cylindrical tuber, large/convex/flat head) have not been previously reported as quality characteristics of raw yam for making boiled yam. Naziha (2002) reported that appearance of tuber and postharvest storability had high importance in the choice of yam varieties while Otoo & Asiedu (2009) stated that the enzymatic oxidation appearance and colour attractiveness of peeled tubers are other main quality characteristics. Barlagne *et al*. (2017) reported that the main important characteristics when buying raw yam were lack of external damage, size, freshness and variety type.

**Table 4:** Quality characteristics of raw yam varieties for making boiled yam of high, intermediate and poor quality as perceived by gender

Source information	High quality		Intermediate quality	Poor quality	
	Men	Women	Skilled women processors	Men	Women
Individual interview and processing diagnosis	*n* = 29	*n* = 57	*n* = 6	*n* = 29	*n* = 57
Smooth skin/without hump or thorns	93	86	67		
Rough/shaggy/humped skin	28	9	83	55	47
Cracked/scratched skin	24	26			
Keeping white or yellow colour during peeling	7	18	83		
Yellow/dark/red/green flesh during peeling				48	35
Ugly tuber				7	23
Presence of rootlets in flesh			50	0	8
Individual interview	*n* = 29	*n* = 51		*n* = 29	*n* = 51
Brown skin	14	24			
Not crumbly skin	7	6			
Thin skin	7	14			
Skin free from holes	10	6			
Not too big or too long tubers	7	4			
Free from rotten odour	3	6			
Big tuber				3	18
Presence of insects				14	0
Processing diagnosis		*n* = 6	*n* = 6		*n* = 6
Large/convex/flat head		100			
Pointed tip		100			
Cylindrical tuber			67		
Flat tuber			83		

### Quality characteristics of yam during processing into boiled yam

The quality characteristics of six yam varieties were evaluated during processing. The main characteristics of the preferred varieties for making boiled yam were as follows: raw tuber appearance (absence of rootlets), ease of peeling, stability of white or yellow colour during peeling and cooking, viscosity of cooking water and the ease of breaking the yam piece with a fork during cooking. The main driver of quality cited by processors is the change of colour into red, purple or dark during peeling, washing/slicing and cooking. Our participatory approach revealed that poor quality characteristics of raw yam for boiling were a sticky substance/mucilage observed during peeling, or the lack of viscous aspect of boiling water. Processors grouped yam varieties into high (Laboko and Kodjewe), intermediate (Gnidou, Kpaina, and Dèba) and poor quality (Kpètè).

### Quality characteristics of boiled yam identified during surveys

Quality characteristics of boiled yam cited by respondents during surveys were classified into sensory (appearance, texture) and emotional (e.g. attractive, unpleasant odour, good aroma/odour, good taste) characteristics (Tables 5). The quality of boiled yams depended on the variety. Poor‐quality boiled yams was mainly referred to as a discolouration (red/purple/black colour) after cooking, poor texture (hard in the mouth/difficult to chew) and/or bad taste/bitter. High‐quality characteristics of boiled yam include appearance, texture when touching, texture in mouth and taste. No significant gender association (chi‐square *P*‐value > 0.05) was evidenced among these characteristics. Prior to consumption, the most important characteristics were a white or yellow boiled yam while easy to break with fork/friable in fingers and sticky were the most important texture characteristics (Table 5). Boiled yam also must have a good aroma and good taste (little sweet/sugary). The ease to chew/friable in mouth is also considered as a high‐quality characteristics for boiled yam. These observations were in agreement with previous work that largely reported the same characteristics of texture, colour, taste, and smell as the major drivers of the consumers' preference for boiled yam (Naziha, 2002; Zannou *et al*., 2007; Ranaivosoa *et al*., 2010).

**Table 5:** Quality characteristics of boiled yam identified during survey (individual interviews, *n* = 80)

Category	Characteristics	Quality descriptors	High quality	Low quality
Sensory	Appearance	White colour	52^a^	
		Yellowish/yellow colour	46	
		Red/green/black colour		32
		Rootlets in the flesh after cooking		7
	Texture to touch	Sticky to the fingers	20	
		Soft to the touch	21	
		Easy to break with fork/friable in fingers	64	
	Texture in mouth	Sticky in mouth	5	
		Soft in mouth	10	
		Easy to chew/tender/friable in mouth	23	
		Hard in mouth/difficult to chew		52
Emotional	Appearance	Pleasant appearance/attractive	56	
	Odour/aroma	Good odour	41	
		Good aroma	17	
		Unpleasant odour		5
	Taste	Good taste	41	
		Bad taste		61

^a^ Numbers indicate per cent of respondents indicating the given classification for each trait.

### Overall liking of boiled yam during consumer testing

To support the high‐ and low‐quality characteristics cited during the survey, boiled yam made from five of the six varieties used during the processing diagnosis were submitted for consumers testing in eight rural communities, one small town and one city. The overall acceptability of boiled yam significantly differed between the five varieties and the locations (rural and urban) (*P* < 0.05) (Table 6). Laboko is the most preferred variety by consumers (score of 7.6, like very much) while Kpètè was scored the lowest (3.7, dislike). Consumers from rural communities gave higher scores for Laboko and Kodjèwé and lower scores for Kpètè boiled yams compared to consumers from Bohicon and Cotonou. The score obtained for Kpètè in the city seemed higher than in the other localities. This indicated that citizens from small town and city seemed to be less able to discriminate the quality differences between boiled yam samples, probably because these consumers are less demanding of specific quality characteristics and are used to consume any types of boiled yam.

**Table 6:** Mean overall liking scores of boiled yam made from five varieties and tasted in rural and urban areas

	Laboko	Kodjèwé	Gnidou	Kpaina	Kpètè
Rural communities (*n* = 129)	7.8^a1^	7.2^b1^	6.0^c1^	6.0^c1^	3.3^d1^
Small town (Bohicon, *n* = 52)	7.4^a2^	7.2^a1^	6.4^b1^	6.2^b1^	3.6^c1,2^
City (Cotonou, *n* = 120)	7.5^a2^	6.7^b2^	6.3^bc1^	6.3^c1^	4.1^d2^
Overall liking score	7.6^a^	7.0^b^	6.2^c^	6.2^c^	3.7^d^

Mean scores with different letters in the same line are significantly different (Tukey's test *P*‐value < 0.05). For each variety in column, means followed with different figures are significantly different (Tukey's test *P*‐value < 0.05).

**Figure 2:**
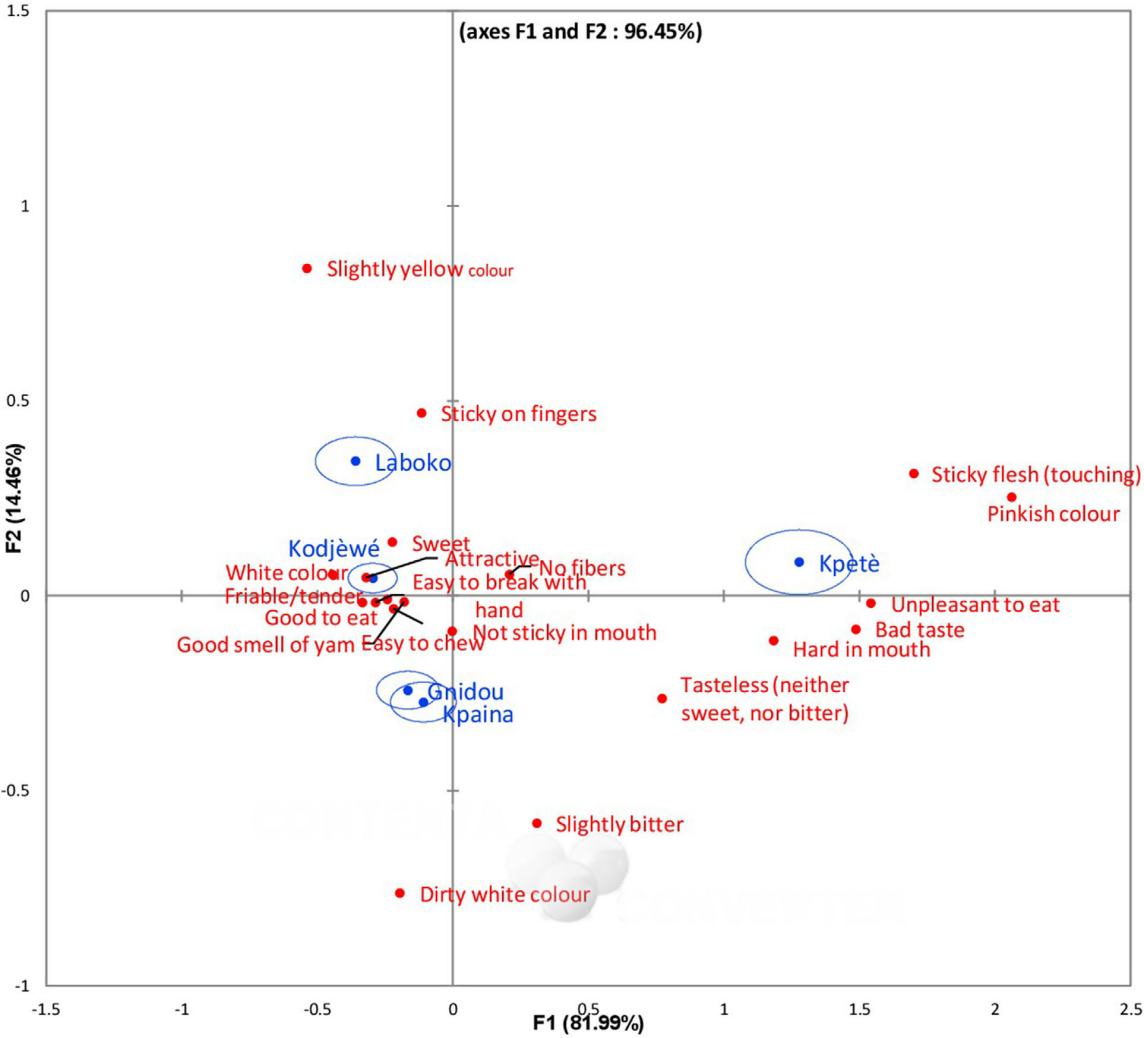
Correspondence factorial analysis of frequency of citations of sensory characteristics selected for Check All That Apply test and five boiled yam varieties. [Colour figure can be viewed at wileyonlinelibrary.com]

The *Q* Cochran's test revealed significant differences in the twenty characteristics used by consumers to describe each boiled yam sample (*P*‐value < 0.05). The most frequent characteristics used to describe the five samples were positive descriptors including attractive, good smell of yam, easy to break with hand, not sticky in mouth, easy to chew, friable/tender, no fibres, sweet and good to eat. Figure 2 depicts a factorial correspondence analysis (FCA) of the five yam varieties and frequency of citations of CATA characteristics selected during surveys and processing diagnosis. Three groups of varieties were established. Boiled yam pieces made from Laboko and Kodjèwé varieties were qualified as high‐quality products and were specifically described as attractive (slightly yellow colour for Laboko and white colour for Kodjèwé), sticky on fingers, no fibres and sweet taste. The varieties considered as intermediate in quality by the consumers during processing diagnosis were Kpaina and Gnidou, both characterised by dirty white colour of boiled samples. Both high and intermediate quality of boiled samples had similar characteristics such as easy to break with hand, not sticky in mouth, easy to chew, friable/tender, good smell of yam and good to eat. Kpètè defined as making poor‐quality boiled yam, was characterised specifically by the pinkish colour, sticky flesh, hard in the mouth, tasteless and unpleasant to eat. This result matches with overall liking data by confirming that Laboko and Kodjèwé boiled yam samples were the most liked (mean score of 7.6 and 7.0, respectively) and Kpètè the least liked (mean score of 3.7). Zannou *et al*. (2007) reported the effect of yam varieties on their processing ability and they argued that boiled yam from Laboko and Kodjèwé had a good taste while Gnidou had a low yam odour. Thus, the sensory characteristics associated with Laboko and Kodjèwé could be considered as the drivers of consumers' liking while the sensory characteristics associated with Kpètè could represent a reason for consumers' rejection.

**Figure 3:**
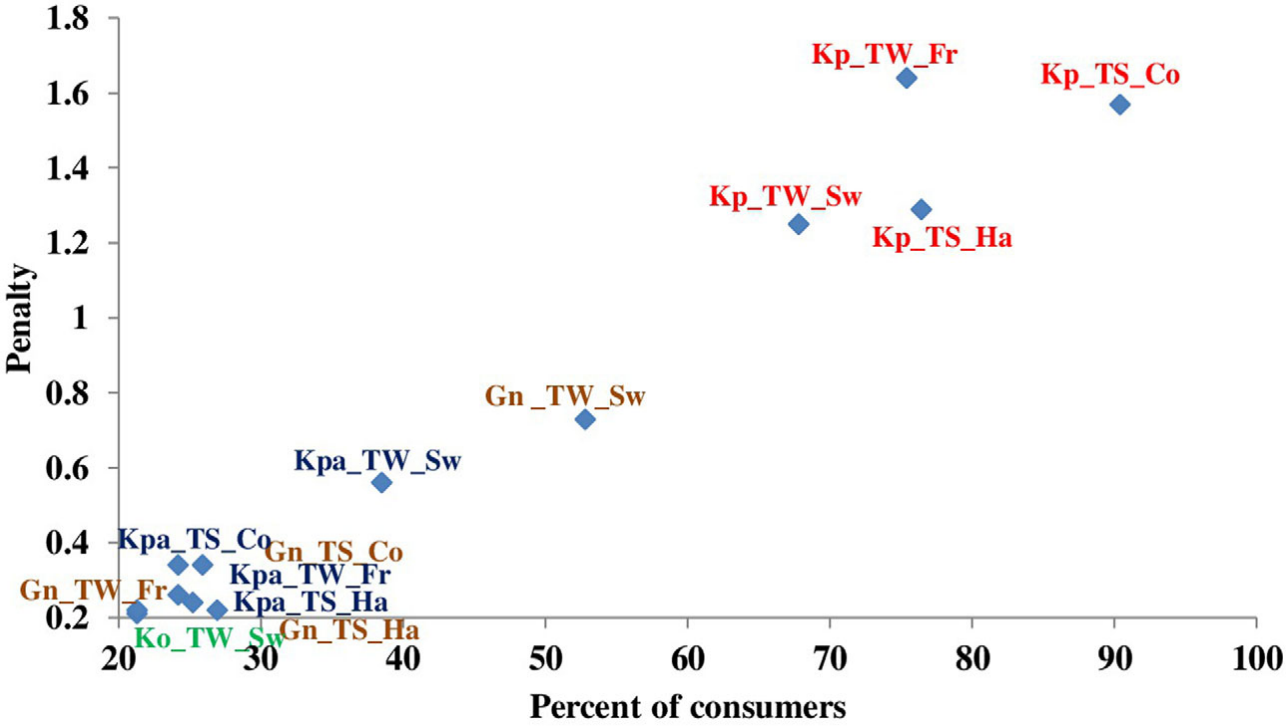
Penalties of overall liking per cent of consumers. Varieties: Laboko (La), Kpaina (Kpa), Gnidou (Gn), Kodjèwé (Ko), Kpètè (Kp) Sensory characteristics: Colour (Co), Friability in mouth (Fr), Hardness in hand (Ha), Sweet taste (Sw) Not‐JAR levels: TW (TW too weak); TS (TS too strong) [Colour figure can be viewed at wileyonlinelibrary.com]

Penalty analysis (Fig. 3) was performed to point out how many scores of overall liking were significantly lost because the characteristic was not evaluated JAR by at least 20% of the consumers. The penalty values of boiled yam ranged from 0.2 to 1.6. The descriptors TS‐colour (*too strong colour i.e. dark)*, TS‐hardness in hand, TW‐sweet taste (too weak *sweet)* and TW‐friability in mouth received the highest mean decreases in the overall liking score (penalty higher than 1.2 and for more than 68% of consumers). The CATA test revealed that the dirty white colour is the main negative characteristic cited by all the consumers to describe Kpaina and Gnidou varieties, but the JAR test showed that this characteristic did not penalise greatly the overall liking. However, both varieties were highly penalised by the TW‐*sweet* taste. This might be explained by the fact that the consumers grant great importance to sweet taste in comparison with dirty colour. This observation was confirmed by the TW‐sweet taste (*not sweet)* cited by 22% of consumers for Kodjèwé, but with the lowest penalty value (0.2). The variety Laboko was considered as the best by processors and consumers and was not penalised by any descriptor evaluated in JAR test. In addition, stickiness in fingers was evaluated JAR by at least 80% of the consumers. Stickiness in fingers did not penalise any product and cannot be considered as a driver of liking.

### Limitations and suggestions

The results obtained are likely to reflect the survey areas, particularly the consumption habit and the yam varieties cultivated. The number and the quantity of yam samples tested during the process diagnosis are likely insufficient in capturing enough detail on quality characteristics, since skilled women processors were asked to process very different varieties, which will invite them to generate a large number of good or bad characteristics throughout the process. It would be interesting to take into account the variance in sensory quality along the yam tuber (proximal, middle and distal) when distributing samples to consumers.

## Conclusions

The quality characteristics of boiled yam motivate the varietal preferences and decisions of producers, processors and consumers. Gender and spatial considerations should be taken into account for yam variety choice/preferences. Field surveys and the processing diagnosis provided reliable descriptors that were mapped with overall liking scores to better understand the consumers' expectations. These consumer expectations can be met by understanding and paying attention to both of the high‐ and low‐quality characteristics of a variety. Three classes of quality characteristics were identified among the six yam varieties studied. Apart from Laboko, and to a lesser extent Kodjewe, all other yam varieties, in particular Kpètè which obtained the lowest score of overall liking, tested by a large number of consumers were penalised for some quality attributes that need to be addressed to biochemists and yam breeders/geneticists.

## Conflict of interest

The authors declare that they have no conflict of interest.

## Author contribution

**Laurenda Honfozo:** Data curation (equal); Formal analysis (lead); Investigation (lead). **Laurent Adinsi:** Data curation (lead); Formal analysis (equal); Investigation (lead); Writing‐original draft (equal). **Alexandre Bouniol:** Conceptualization (equal); Investigation (equal); Software (equal). **Sounkoura Adetonah:** Investigation (equal); Project administration (equal); Supervision (equal). **Lora Forsythe:** Conceptualization (equal); Methodology (lead); Project administration (equal). **Ulrich Kleih:** Methodology (equal); Validation (equal). **Joseph D. Hounhouigan:** Project administration (equal); Supervision (equal); Validation (equal); Writing‐original draft (equal). **Geneviève Fliedel:** Conceptualization (equal); Formal analysis (supporting); Methodology (equal); Validation (equal); Writing‐original draft (equal). **Noël H. Akissoe:** Formal analysis (equal); Investigation (equal); Project administration (lead); Supervision (lead); Writing‐original draft (lead).

## Ethical assessment and consent

This study was assessed and approved by the ‘Comité National d'Ethique pour la Recherche en Santé’ of Benin and the CIRAD Ethics Committee. Samples were prepared according to good hygiene and manufacturing practices. Participants were informed about the purpose of the study and explained that their participation was entirely voluntary, that they could stop the interview at any point and that the responses would be anonymous. Written consent (signature) was sought from interviewers and consumers participating in this study.

### Peer Review

The peer review history for this article is available at https://publons.com/publon/10.1111/ijfs.14707.

## Data Availability

The data that support the findings of this study are available on request from the corresponding author. The data are not publicly available due to privacy or ethical restrictions.
